# Supplementary material for: Effect of CO2 Content in Air on the Activity of Carbonic Anhydrases in Cytoplasm, Chloroplasts, and Mitochondria and the Expression Level of Carbonic Anhydrase Genes of the α- and β-Families in Arabidopsis thaliana Leaves
Source: Plants (Basel). 2022 Aug 14;11(16):2113. doi: 10.3390/plants11162113 (PMC9414674; doi:10.3390/plants11162113)
Supplement: Supplementary file 1 [file plants-11-02113-s001.zip › plants-1797641-supplementary.pdf]

Supplementary Material

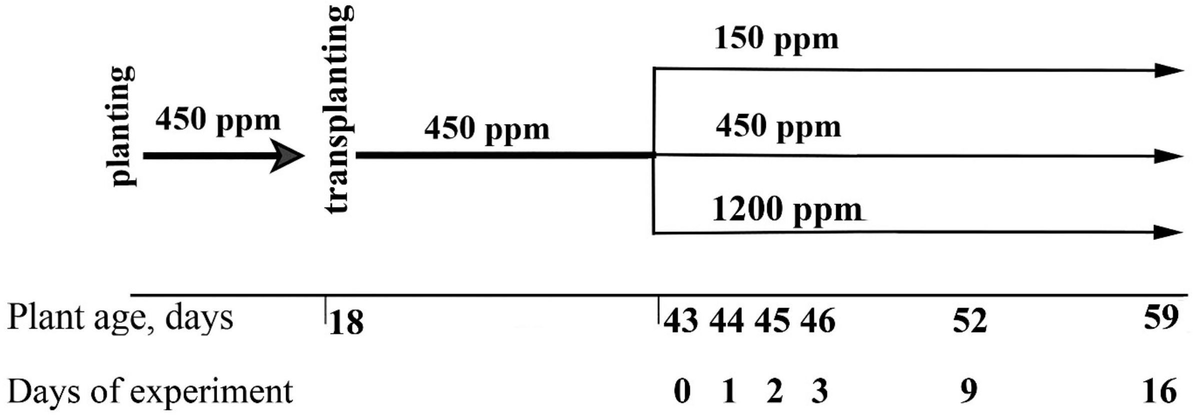

Figure S1. Scheme of *Arabidopsis* plants cultivation during experiment. Plants were grown at CO_2 content of 450 ppm, temperature 19°C, 8 h day/16 h night photoperiod, photosynthetically active radiation (PAR) of 50–70 µmol quanta m^{-2} s^{-1} until 6 weeks of age and then exposed to conditions of the low and high CO_2 content, 150 and 1200 ppm for 16 days.

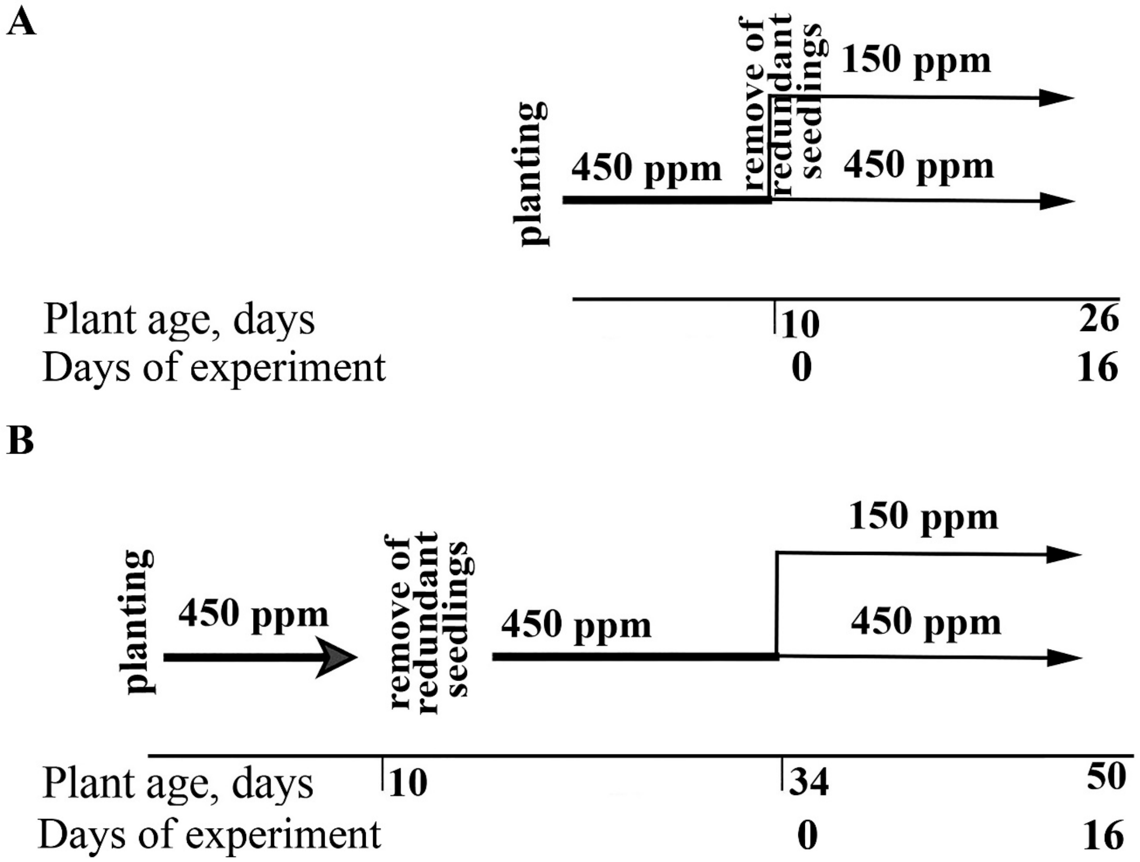

Figure S2. Scheme of *Arabidopsis* plants cultivation during experiment on the effect of the low CO_2 level on the expression of CA genes. Plants were grown at CO_2 content of 450 ppm, temperature 19°C, short day photoperiod 8 h day/16 h night, photosynthetically active radiation (PAR) of 50–70 µmol quanta m^{-2} s^{-1} until 10 (A) and 34 (B) days of age and then exposed to conditions of the low CO_2 content of 150 ppm for 16 days.

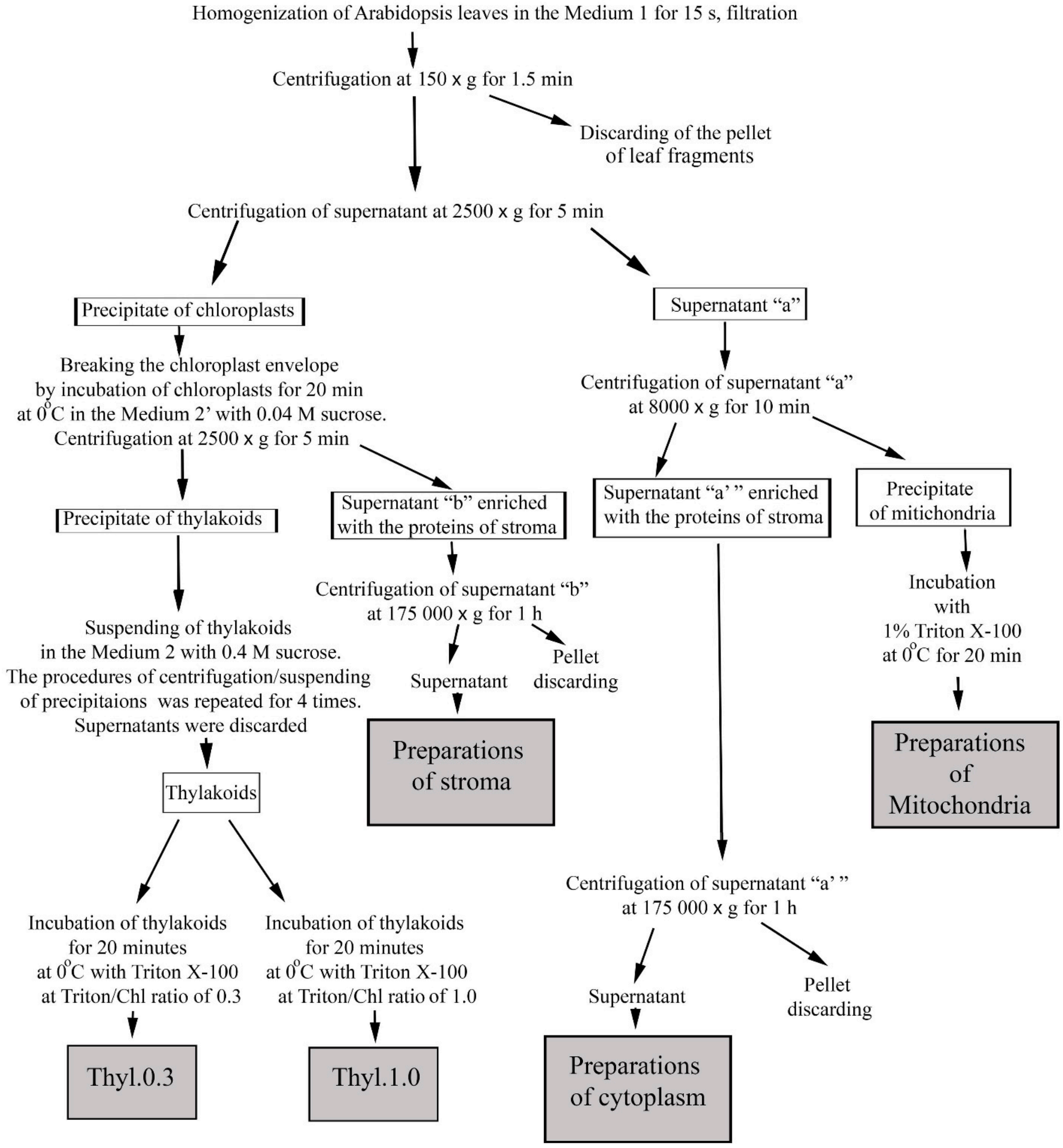

Figure S3. Scheme of isolation from Arabidopsis leaves of preparations of cytoplasm, mitochondria, stroma and thylakoids. Fractions obtained during isolation are framed, preparations in which CA activity was determined are highlighted in gray.
